# Supplementary figures and images for: Whole genome sequencing analysis identifies recurrent structural alterations in esophageal squamous cell carcinoma
Source: PeerJ. 2020 Jun 26;8:e9294. doi: 10.7717/peerj.9294 (PMC7323713; doi:10.7717/peerj.9294)

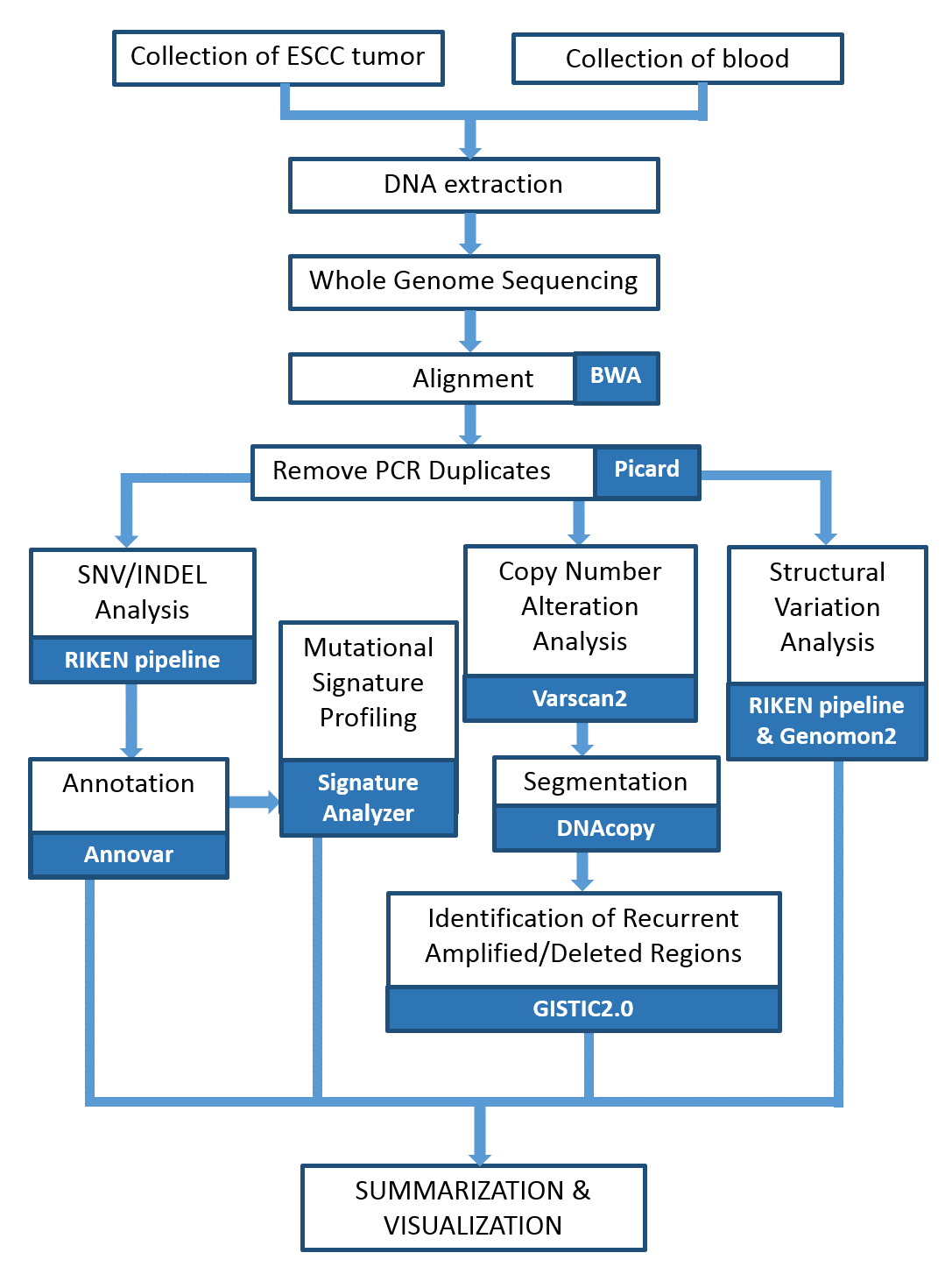

Supplement: Figure S1 — We performed WGS on 20 ESCC samples. BWA was used for the alignment with the reference genome. We used Riken-in-house pipeline to call the SNVs/INDELs. For mutational signature profiling, we used SignatureAnalyzer. To call the somatic CNAs from the WGS of ESCC samples, we used Varscan2. DNAcopy was used to do the segmentation. For the Structural Variation analysis, we used Riken-in-house pipeline and Genomon2. Finally, merged both the SVs list in order to identify distinct SVs in ESCC. [file peerj-08-9294-s001.png]

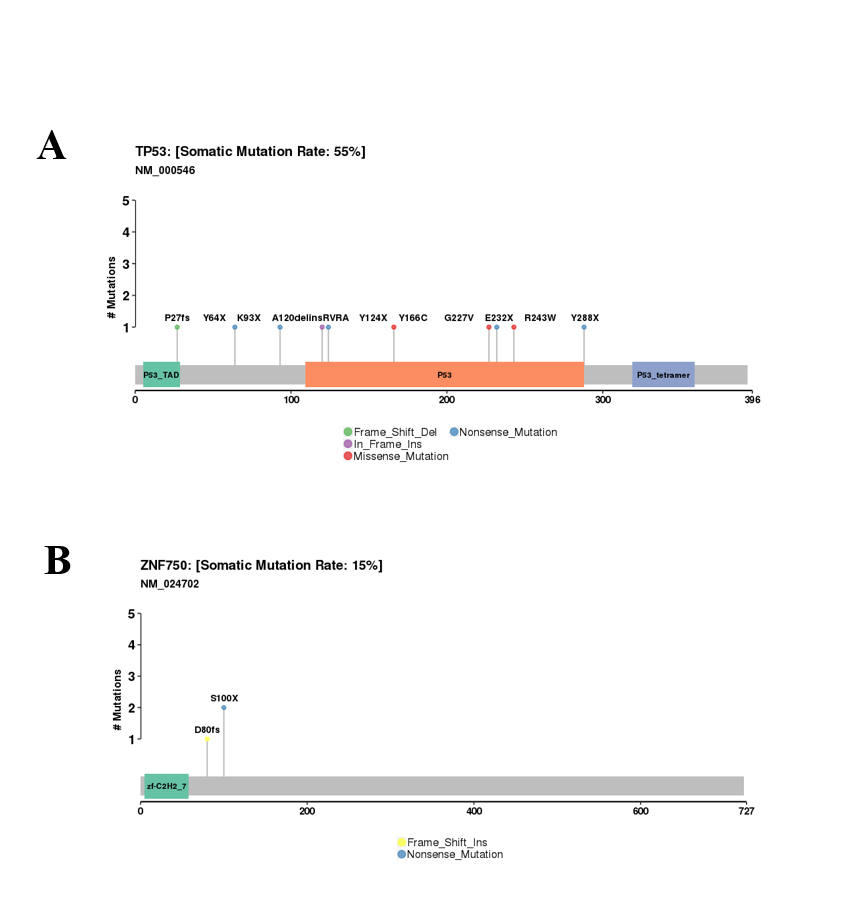

Supplement: Figure S2 — Distribution of TP53 (A) and ZNF750(B) mutations in ESCC. P53_TAD, transactivation domain; P53, DNA-binding domain; P53_tetramer, tetramerization domain. [file peerj-08-9294-s002.png]

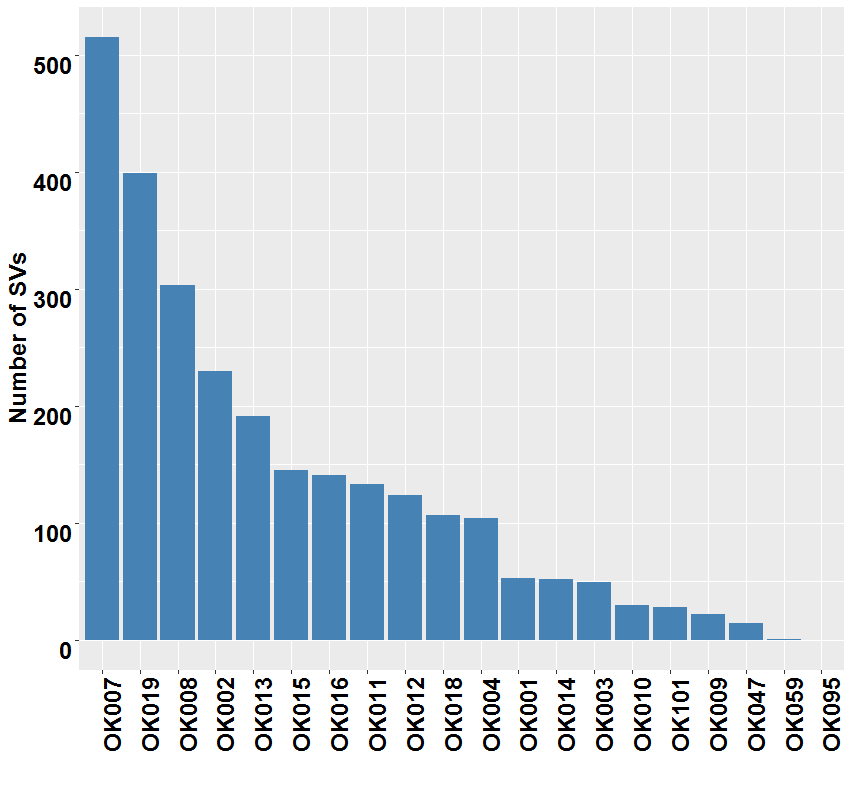

Supplement: Figure S3 — The figure visually shows the SVs affect most chromosomes in OK007 and OK008. [file peerj-08-9294-s003.png]

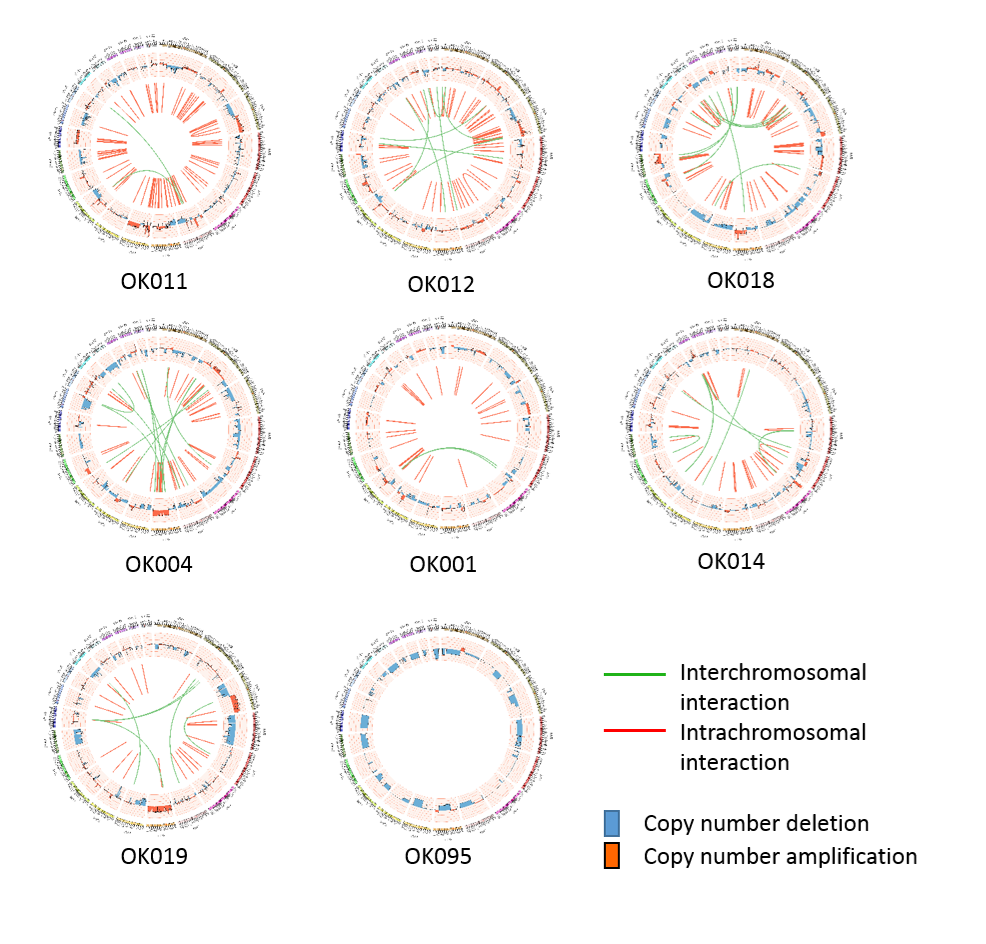

Supplement: Figure S4 — The inner ring represents the SVs: red for intrachromosomal interactions, and green for interchromosomal interactions. The second ring next to SVs displays the CNAs: red for amplifications and blue for deletions. The outer ring shows the chromosome ideogram. [file peerj-08-9294-s004.png]

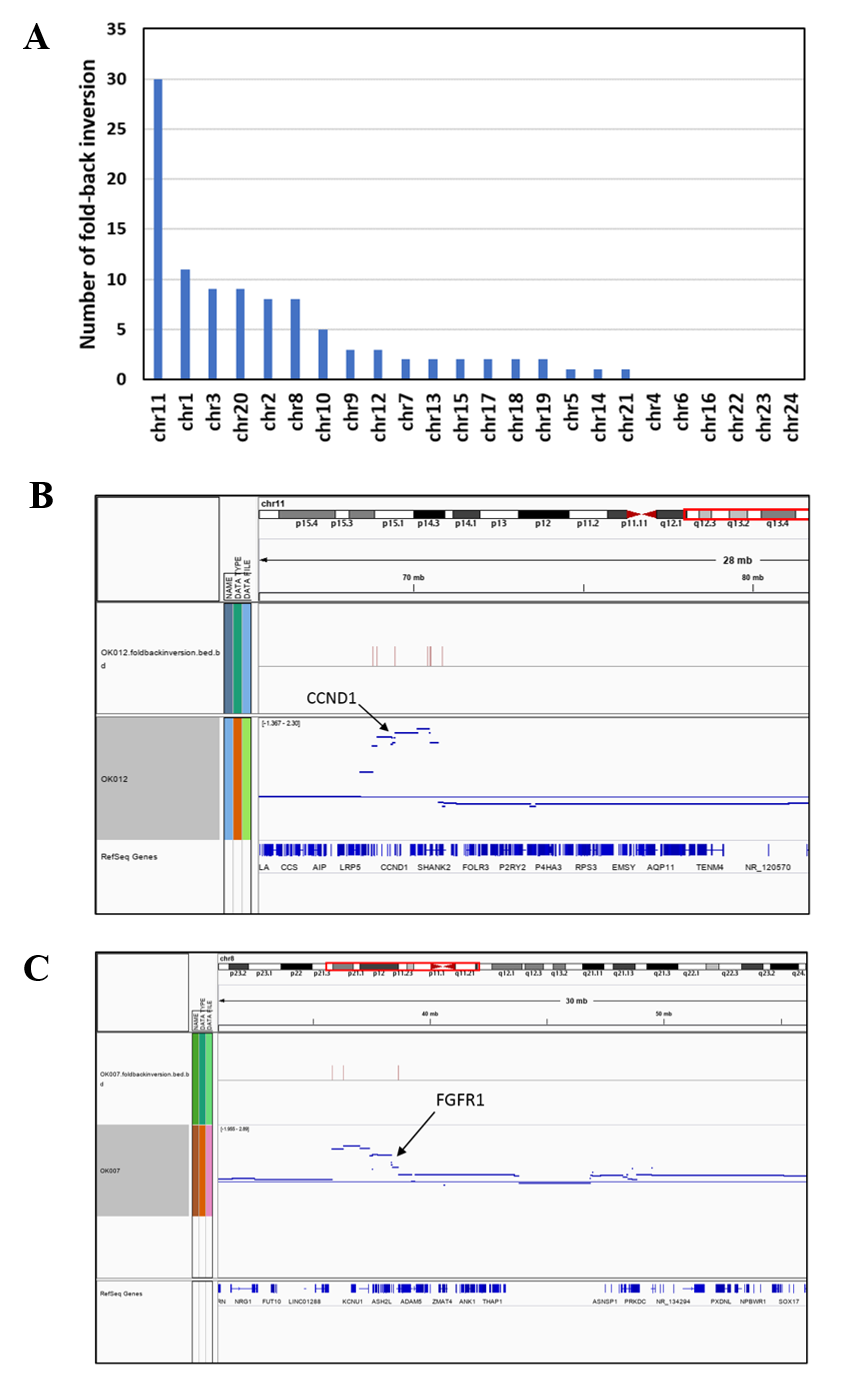

Supplement: Figure S5 — (A) The number of fold-back inversions identified in 20 ESCC patients. (B) The figure represents the amplification of CCND1 due to the BFB effect in one ESCC sample, and (C) shows amplification of FGFR1 as a result of BFB in another sample. [file peerj-08-9294-s005.png]

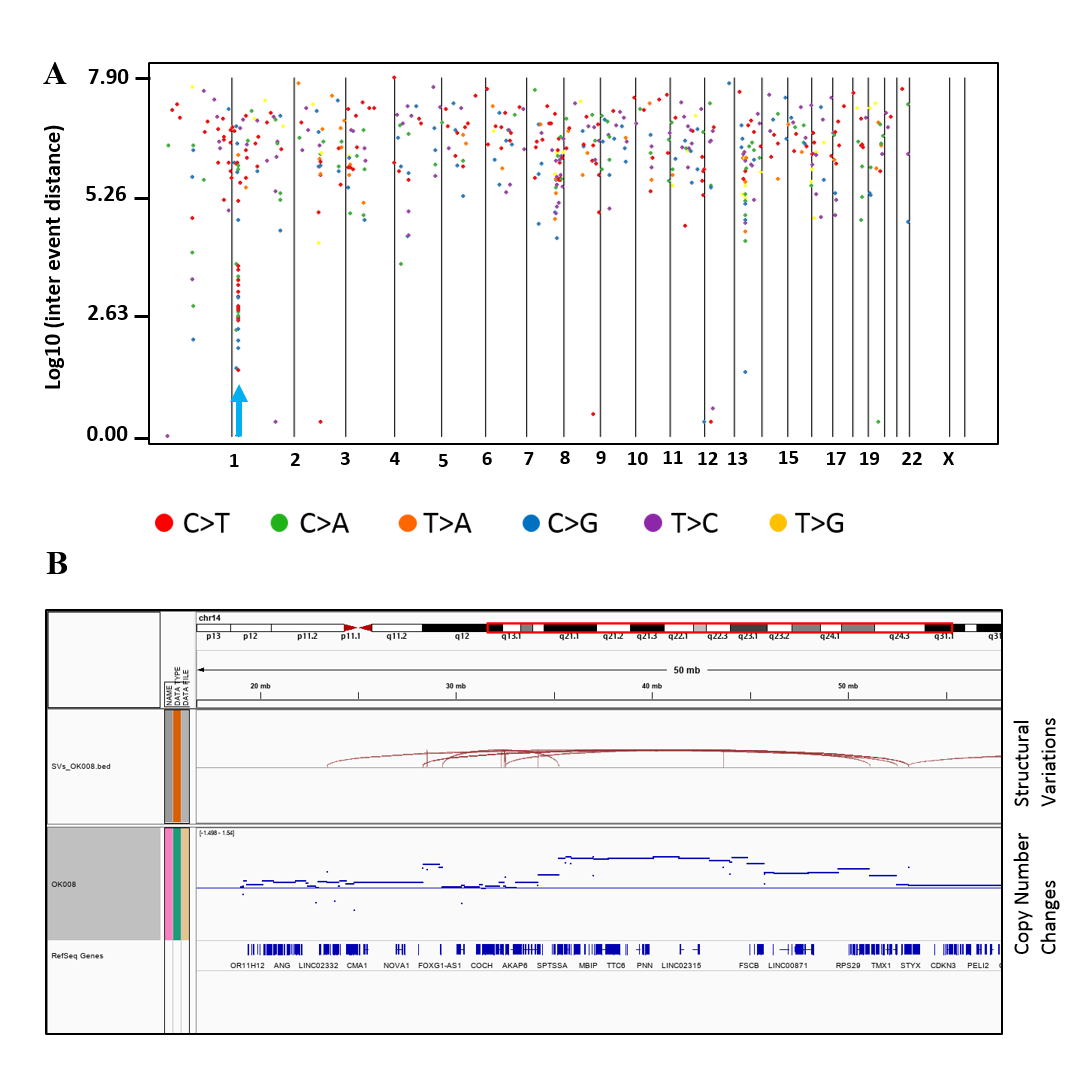

Supplement: Figure S6 — (A) In the rainfall plot, each dot represents a single somatic mutations. The X-axis shows the chromosomes in order and Y-axis shows the inter-genomic distance of the mutations. The blue cursor indicates the position of hyper-mutation on chromosome 1. (B) Chromothripsis on chromosome 14 in one ESCC patient. The cluster of SV breakpoints in the middle panel (red bars) present in conjunction with regular copy number gains in the bottom panel. [file peerj-08-9294-s006.png]
